# Predictive Vascular Changes in OCTA in Diabetic Patients

**DOI:** 10.3390/biomedicines13061486

**Published:** 2025-06-17

**Authors:** Jelena Cuk, Dejana Stanisavljevic, Jelena Vasilijevic, Milica Jeremic Kaplarevic, Milica Micovic, Aleksandar Risimic, Dijana Risimic

**Affiliations:** 1 Clinic for Eye Disease, University Clinical Centre of Serbia, 11000 Belgrade, Serbia; 2 Faculty of Medicine, University of Belgrade, 11000 Belgrade, Serbia; 3 Institute for Medical Statistics and Informatics, Faculty of Medicine, University of Belgrade, 11000 Belgrade, Serbia

**Keywords:** diabetic retinopathy, OCT angiography, vascular density

## Abstract

**Background/Objectives**: The aim of this study was to investigate quantitative differences in optical coherence tomography angiography (OCTA) between diabetic patients and healthy controls and to identify the early OCTA biomarkers for diabetic macular changes. **Methods**: Ophthalmological examination and OCTA were performed on two groups of diabetic patients (with and without mild diabetic retinopathy) and healthy controls. Macular, foveal, perifoveal, and parafoveal vessel density (VD) in the superficial capillary plexus (SCP) and deep capillary plexus (DCP), foveal avascular zone (FAZ), and flow area in the choriocapillaris were calculated. **Results**: A total of 431 eyes of 233 participants were analyzed. The VD in the SCP in the whole macula was the lowest in the DM + DR group and lower than in the DMnoDR group; however, in the fovea, it was the highest in the DM + DR group and higher than in the DMnoDR group. The VD in the SCP in the parafovea was lower in the DM + DR group than in the DMnoDR group, and in the perifovea, it was lower in the DMnoDR group than in the control group. The VD in the DCP in the macula, parafovea, and perifovea was lower in the DM + DR group than in the DMnoDR and control groups. The FAZ and flow areas in the choriocapillaris were smaller in the DM + DR group than in both the DMnoDR and control groups. **Conclusions**: VD reduction in the SCP and the DCP of the macular and parafoveal regions, as well as in the DCP of the perifoveal region, may indicate progression of diabetic retinopathy from subclinical to clinical stages; however, an increase in the foveal region in the SCP can be a compensatory mechanism. VD reduction in the perifovea and whole macula in the SCP can be a screening factor for subclinical macular changes. FAZ reduction before clinical signs of retinopathy may be an early compensatory vascular mechanism.

## 1. Introduction

Diabetic retinopathy (DR) is the most common microvascular complication of diabetes mellitus (DM) and is a leading cause of blindness worldwide [1]. Chronic hyperglycemia, as well as consequential inflammation and hypoxia, leads to numerous microvascular changes, such as loss of pericytes and endothelial cells, which result in increased vascular permeability, occlusion, ischemia, and neovascularization. All of the above make diabetic retinopathy a degenerative neurovascular disease, the early detection of which is of crucial importance for timely therapy.

Optical coherence tomography angiography (OCTA) is a new noninvasive imaging method for the visualization of retinal and choroidal vascularization using the movements of erythrocytes through blood. OCTA generates multiple and repeated OCT B-scans at the same location and detects differences in the reflectivity of blood cells, which allows separate evaluation of microvascular changes in superficial, intermediate, and deep capillary plexuses.

OCTA technology uses the split-spectrum amplitude-decorrelation angiography (SSADA) algorithm to detect blood flow in the retinal tissue. Image areas of 3 mm × 3 mm or 6 mm × 6 mm can be obtained. After obtaining the retinal OCTA image, segmentation can be performed to visualize individual retinal vascular layers separately. The AngioVue software ( version 2017.1.0.151) segments OCTA images into separate retinal vascular layers—the superficial capillary plexus (SCP), which corresponds to the vasculature within the ganglion cell layer, and the deep capillary plexus (DCP), which corresponds to the vasculature on either side of the inner nuclear layer (INL) [2].

In addition to the qualitative analyses, which include evaluation of foveal avascular zone shape, the presence of non-perfusion areas, microaneurysms, new vessels, etc., OCTA also enables quantitative analyses, such as measurements of foveal avascular zone area (FAZ) and vessel density (VD), both in SCP and DCP.

Vessel density was defined as the percentage area occupied by vessels [3]. The foveal avascular zone is the capillary-free area encircled by the foveal capillary ring in the center of the macula [4]. The dimension of the FAZ area is larger in diabetic patients and increases with the progression of diabetic retinopathy [5,6,7].

The aim of our study was to investigate quantitative differences in OCTA between diabetic patients and healthy controls and to identify the early OCTA biomarkers for diabetic macular changes.

## 2. Materials and Methods

Our cross-sectional observational study was conducted in the Clinic for Eye Diseases, University Clinical Centre of Serbia. Participants were consecutively recruited between June and September 2024. All included patients who were diagnosed with type 2 diabetes mellitus (DM2) were referred by an endocrinologist for a follow-up ophthalmological examination and had no vision-related complaints. All the participants signed an informed consent allowing the use of their data in the research. Demographic data (patient’s age, gender) and data on duration of DM, type of therapy (oral antidiabetics/insulin), hypertensive status, and the value of the most recent HbA1C (not older than one month) were collected. Hypertensive status was determined based on whether the patients were on oral antihypertensive therapy and whether their condition was controlled. Their most recent blood pressure value, not older than 24 h, was requested. When collecting data on the duration of the disease, the onset of the disease was considered the moment when the disease was diagnosed by a doctor (endocrinologist or primary care physician).

All the subjects were then examined by two independent ophthalmology specialists. Clinical examination of the anterior and posterior segments of the eye was performed. All patients with diabetic macular edema or signs of diabetic retinopathy classified as stage > 35 based on the ETDRS grading system were excluded from the study. Additionally, patients who had any other associated eye diseases (active uveitis, keratitis, or any other inflammation, glaucoma, cataract impairing the optical media transparency, and other macular diseases such as senile macular degeneration, macular dystrophies, ocular vascular occlusive diseases, and other retinal conditions) were excluded from the study. The remaining patients were divided into two groups: those who had no diabetic retinopathy changes (ETDRS level 10), referred to in further text as the DMnoDR group, and those who had microaneurysms (ETDRS 20), hemorrhages, hard exudates, and cotton wool spots (ETDRS 35), referred to in further text as the DM + DR group.

The control group consisted of healthy subjects who did not have DM (all presented HbA1C and blood glucose levels not older than one month) and who visited the Clinic for correction of a refractive anomaly (no greater than ±5 diopters) and/or cataract screening. These participants also signed an informed consent form prior to participation in the study, and data on their age and gender were collected. Additionally, they were all asked to provide data about their hypertension status, similar to the diabetic participants. Subsequently, an ophthalmological examination was performed, and the participants were excluded from the study based on the same criteria mentioned above regarding associated eye diseases.

**Figure 1:**
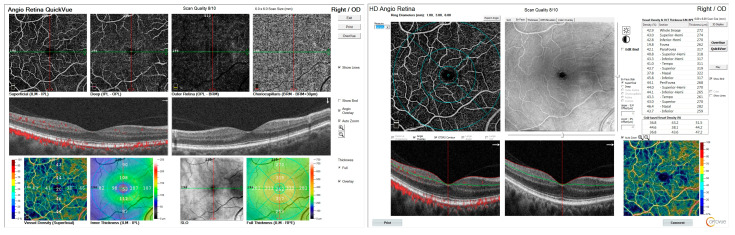
Distribution of vessel density values using AngioVue Analytics software.

Second to clinical examination, the best-corrected visual acuity (BCVA) was recorded, and the intraocular pressure of all participants was recorded. The BCVA was 1.0 (Snellen equivalent: 20/20) in all participants. Subjects who did not achieve a BCVA of 1.0 for any reason were excluded from the study. OCTA was then performed on all subjects in all three groups. An AngioVue OCTA device (Optovue, Inc., Fremont, CA, USA) using the SSADA algorithm was used to obtain angiography images. OCTA scans were acquired over 6 × 6 mm regions using a 70 kHz OCT system with a scan pattern consisting of two repeated B-scans at 304 raster positions, with each B-scan comprising 304 A-scans. The flow signal was detected by analyzing consecutive scans of the same area and comparing differences, or decorrelations, between them. Scans with low quality (i.e., presence of blink or motion artifacts) were repeated until good quality scans were obtained (signal strength index above 60). Images that did not reach these values even after five repetitions were excluded from further analysis. Automated segmentation was used to define superficial capillary plexus (SCP), deep capillary plexus (DCP), and choriocapillaris (CC). The SCP was defined as the region extending from 3 µm beneath the internal limiting membrane (ILM) to 15 µm below the inner plexiform layer (IPL). The DCP was delineated as the area between 15 µm and 71 µm beneath the IPL. Vessel density (VD) was quantified using the built-in AngioVue Analytics software, which generates a binary map of the vasculature within the specified region (Figure 1). The foveal region was defined as the central ring with a radius of 0.3 mm. Parafoveal vessel density was assessed within a ring-shaped region extending from 0.3 mm to 1.25 mm in radius from the center of the macula, while perifoveal vessel density was measured in the annular zone between 1.25 mm and 2.75 mm from the center of the fovea.

Central macular thickness (CMT) is defined as the vertical distance between the ILM and Bruch’s Membrane (BM) in the central part of the macula (fovea). A grid defined by ETDRS standards was used, where the central 1 mm diameter circle represents the zone for measuring CMT. The software calculates the average retinal thickness in this region. Diabetic macular edema (DME) was defined as a CMT of more than 300 μm. These eyes were excluded from further analysis.

The FAZ area in SCP is calculated automatically by the AngioVue software using boundary delineation of the capillary-free zone in the en face image. The measurement is provided in square millimeters (mm^2^).

The flow area in the choriocapillaris (flow CC) is measured within the entire CC region located 30 µm beneath the retinal pigment epithelium (RPE), where OCTA detects blood flow. A circular area of 3.144 mm^2^ centered on the fovea is automatically defined by the software. Within this region, the software calculates the total area (in mm^2^) of multiple high-signal regions corresponding to the CC lobular meshwork, where blood flow is visualized by the OCT angiography system. The measurement is expressed in square millimeters and reflects the portion of the CC in which flow is detected within the 3.144 mm^2^ circular region.

All participants’ eyes were included in the study if they met the criteria. Data on the VD in the entire macula, fovea, parafoveal, perifoveal regions, FAZ area, and flow CC were collected from the OCTA scans.

### Statistical Analysis

All statistical analyses were performed using SPSS software version 26 (SPSS, Inc., Chicago, IL, USA). Categorical variables were compared using the chi-square test. The Shapiro–Wilk normality test was used to test the normality of the data. One-way ANOVA and post hoc test of multiple comparisons were used for parametric comparisons, and Kruskal–Wallis and Mann–Whitney tests were used for non-parametric comparisons of the FAZ area. *p*-values less than 0.05 were considered statistically significant.

## 3. Results

### 3.1. General Characteristics

A total of 431 eyes belonging to 233 participants were analyzed. The DMnoDR group contained 91 patients and 175 eyes, the DM + DR group contained 45 patients and 84 eyes, and the control group contained 97 participants and 171 eyes.

The mean age was 66.56 ± 9.4 years (37–87); participants in the DMnoDR group had a mean age of 65.42 ± 10 years, the DM + DR group had a mean age of 66.86 ± 9.3 years, and the control group had a mean age of 67.51 ± 8.7 years. There were no significant differences in age between the three groups (*p* = 0.323).

There were a total of 135 females (57.9%) and 98 males (42.1%) in the study. The mean duration of type 2 diabetes mellitus was 8.2 ± 0.8 years, with 9.9 ± 8 in the DMnoDR group and 16.7 ± 6.6 in the DM + DR group; the median duration was 10 years (0–40) in the DMnoDR group and 15 years (15–30) in the DM + DR group. HbA1C levels were 8.8% ± 2.6 in the DMnoDR group and 8.9% ± 2 in the DM + DR group.

The difference in disease duration between the DM + DR group and the DMnoDR group was statistically significant (*p* < 0.001), with a longer disease duration in the DM + DR group.

The prevalence of hypertension was not significantly different between groups because almost all participants had oral hypertensive therapy. Hypertension was under control in all three groups.

### 3.2. OCTA Parameters

Table 1 and Table 2 show VD in the SCP and the DCP, respectively. Table 3 shows the flow CC and the FAZ area.

**Figure 2:**
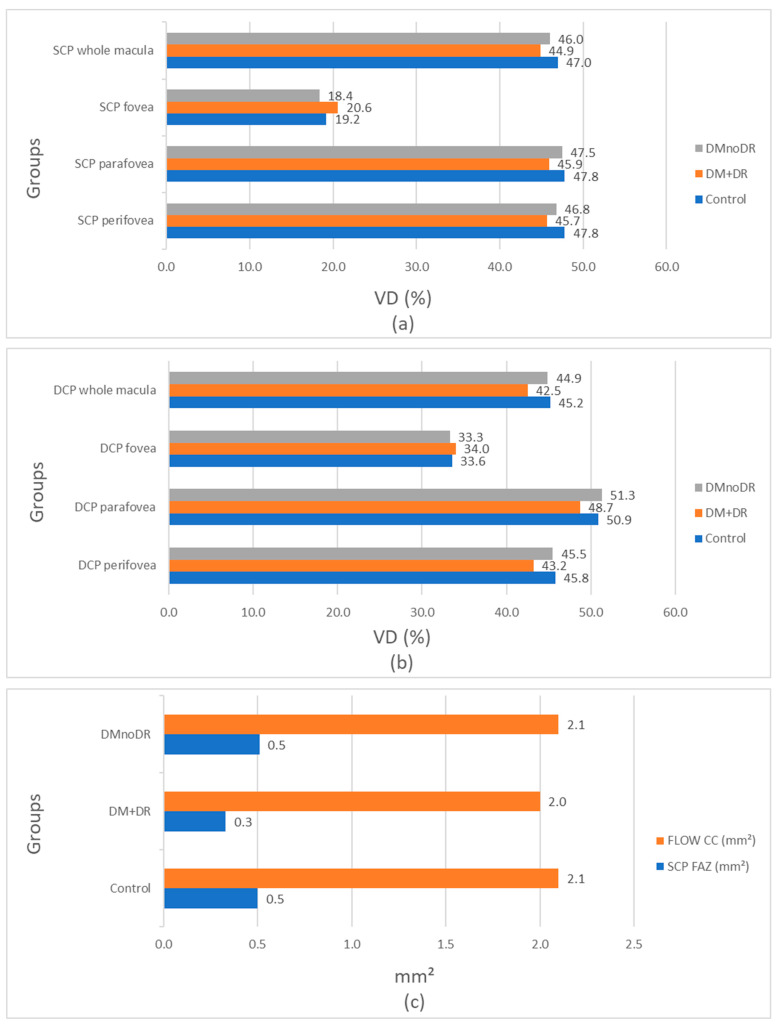
Charts of vascular density changes in the superficial capillary plexus (**a**) and deep capillary plexus (**b**), and of the flow area in the choriocapillaris and foveal avascular zone (**c**) from healthy and diabetic eyes.

As shown in Figure 2a, there is a progressive reduction in vascular density in the superficial capillary plexus (SCP) from healthy individuals to diabetic patients with retinopathy in the whole macula and the perifoveal and parafoveal regions. However, in the foveal region, vascular density is the highest in the DMnoDR group.

Figure 2b shows a similar pattern of VD reduction in the whole macula and the peri-foveal regions of the deep capillary plexus (DCP), while in the fovea, VD is again the highest in the DMnoDR group, although this is not statistically significant.

Figure 2c shows that both the flow CC and the FAZ area in SCP are smallest in the DM + DR group.

## 4. Discussion

Our results show that, when observing the whole macular image in the superficial capillary plexus, VD was statistically significantly the highest in the control group, followed by the DMnoDR group, and the lowest in the DM + DR group. This observation of progressive VD reduction—from healthy controls to diabetic patients without retinopathy and then to those with retinopathy—aligns with the results of numerous other studies. For example, Ghassemi reported a consistent pattern of VD decrease in the SCP from healthy individuals to non-DR diabetic patients and further to those with non-proliferative diabetic retinopathy [8]. In general, most studies have shown that VD decreases with the progression of diabetic retinopathy, with some reporting statistically significant results [9,10,11,12,13,14,15] and others non-significant results [16].

Analyzing the three macular regions in the SCP separately gave different results. VD in the foveal region of the SCP was the highest in the DM + DR group and the lowest in the DMnoDR group, which is an unexpected finding. Comparisons between groups revealed a statistically significant difference only between the DM + DR and DMnoDR groups (in favor of the DM + DR group), highlighting potential changes occurring in the early stages of the disease. It is important to understand the implications of this result and to clarify whether the observed increase in vascular density in the fovea reflects compensatory capillary recruitment [17], inner retinal thinning and projection artifacts [18,19], foveal sparing of capillary dropout, or the influence of inflammatory and angiogenic factors [20], and whether it can serve as a potential biomarker of the disease.

Furthermore, several other studies have reported increased foveal VD in the early stages of diabetic retinopathy (DR) or in cases of mild non-proliferative diabetic retinopathy (NPDR). For example, Li et al. found higher foveal VD in patients with mild NPDR compared with those without DR, suggesting early vascular reactivity or compensatory mechanisms [21]. Other studies also reported increased foveal VD in diabetic patients with mild retinopathy compared with those without [22].

We obtained results similar to those observed in the whole macula in the parafoveal region (closer to the fovea) of the macula within the SCP: VD was the lowest in the DM + DR group and significantly lower than in both control and DMnoDR groups. These findings demonstrate a decrease in VD in the parafoveal region with the onset of clinically visible retinopathy (ETDRS levels 20 and 35). However, VD in the parafoveal region of the SCP does not appear to be a predictor of early diabetic changes, as the difference between the healthy controls and DMnoDR groups was not statistically significant. Dimitrova et al. reported a significant decrease in parafoveal vascular density in diabetic patients without retinopathy compared with controls, but only in the DCP, not in the SCP [23].

In the perifoveal region of the SCP, VD was the highest in the control group and significantly higher than in both diabetic groups. Additionally, when comparing the two diabetic groups, VD was higher in the DMnoDR group than in the DM + DR group, although this difference was not statistically significant. Polat Gültekin and Hamurcu reported that VD values in the SCP were significantly lower in the whole macula, fovea, and perifovea—but not in the parafoveal area—in diabetic patients without retinopathy compared with healthy controls [24].

VD in the DCP within the macular region cannot be considered a predictor of early vascular changes in diabetes in our patients. Unlike in the SCP, VD in the foveal region of the DCP did not differ significantly between the three groups, although the highest VD was observed in the DM + DR group, similar to the pattern observed in the SCP. The decrease in VD in the DM + DR group compared with the control group was statistically significant in the whole macula and the parafoveal and perifoveal regions, but not in the fovea. Additionally, the VD in the whole macula and the parafoveal and perifoveal regions was significantly higher in the DMnoDR group than in the DM + DR group, indicating that these parameters may reflect more advanced vascular changes at the stage when retinopathy becomes clinically detectable.

Although VD in the fovea of the DCP was higher in the DM + DR group than in the DMnoDR group, the difference was not statistically significant. Nevertheless, this may suggest a similar pattern of increased vascular density in the fovea during the early stages of clinically visible retinopathy in the DCP, as observed in the SCP. Simonett et al. reported reduced parafoveal VD in the DCP among patients with mild diabetic retinopathy (type 1 DM) compared with controls [25]. In contrast, Polat and Hamurcu found no difference in the VD in the DCP between healthy controls and the DMnoDR group [24].

Some previously mentioned studies [8] demonstrated a trend toward a lower median vessel density (VD) from healthy subjects to patients with proliferative diabetic retinopathy (PDR), not only in the macular area but also across various subregions. This trend was evident in both the SCP and DCP, with the exception of foveal VD in the SCP, which was lower in the group without retinopathy than in the groups with retinopathy and healthy controls, although this difference was not statistically significant. A continuous and significant decrease from healthy individuals to the NPDR group was observed in both the SCP and DCP [8]. Similarly, Ryu et al. showed that vessel density in both the SCP and DCP decreased with increasing DR severity in the parafoveal and perifoveal regions [26]. In contrast, our results indicate that VD in the whole macula and the parafoveal region, in both the SCP and DCP, could serve as a potential indicator of disease progression.

Hwang reported a 12.6% reduction in parafoveal VD and a 10.4% reduction in perifoveal VD in eyes with DR compared with healthy controls, suggesting that OCTA may be able to differentiate DR eyes from non-diabetic eyes [27]. Other investigators found that the perfusion reduction was significantly more pronounced in the DCP than in the SCP, and in the perifoveal region compared with the parafoveal region [3]. Similarly, several studies have shown a progressive decrease in VD at the parafovea (defined as the area between an inner 1 mm ring and an outer 2.5 mm or 3 mm ring centered on the fovea) in both the SCP and DCP as DR severity advances [28,29].

Several studies have shown that differences in capillary density between DR stages are detected more frequently in the SCP than in the DCP, as fewer parameters reach statistical significance among DCP subgroups compared with those of the SCP [13]. Nevertheless, Nesper et al. reported that VD in the DCP correlated most strongly with DR severity [28]. This apparent contradiction may be explained by the fact that capillary loss in the DCP is particularly consequential: the deep plexus supplies the outer plexiform layer, which has high metabolic demands and is especially vulnerable to ischemic damage.

Taking everything into account, VD reduction in the SCP of the macular and parafoveal regions in our study may be an indication of disease deterioration from clinically invisible to clinically visible retinopathy; however, VD increase in the foveal region can be a compensatory mechanism. VD reduction in the perifovea and whole macula in SCP can be used to screen for diabetic patients without retinopathy. Diabetic patients with mild retinopathy have a reduction in VD in the macular, parafoveal, and perifoveal regions in the SCP, but not in the foveal region, which can be the result of an early compensatory mechanism in the fovea because of very mild diabetic retinopathy changes (ETDRS 20 and 35). VD in DCP is not a screening factor for diabetic patients without retinopathy in any macular region. VD in DCP in macula, perifovea, and parafovea regions is significantly lower in diabetic patients with retinopathy than in diabetic patients without retinopathy; thus, these three parameters could be predictors of developing diabetic retinopathy from subclinical to clinical form.

The flow area (or perfusion area) in the CC was lowest in the DM + DR group, and it was lower than both the control and DMnoDR groups. This difference was statistically significant and suggests that diabetic patients with mild non-proliferative retinopathy have decreased flow in the CC, as they also have decreased VD in the SCP and DCP. Furthermore, as we have shown that decreased VD in the macular, perifoveal, and parafoveal regions of the SCP and DCP, along with increased VD in the foveal region of the SCP and DCP, could be a potential predictive factor for the transition from subclinical retinopathy to clinically visible retinopathy, the same applies to decreased flow in the CC. Many other studies confirm our results [29,30,31,32]. For example, perfusion density in the CC is significantly lower in the proliferative diabetic retinopathy (PDR) group than in non-DM controls and diabetic patients without DR [30]. Additionally, decreased CC flow was observed in the area overlying the parafovea in PDR and NPDR patients compared with non-DM controls, but no changes were observed in CC flow in patients with DM without DR [31]. However, other studies have found significantly lower flow in the fovea of the CC in DM subjects without DR compared with non-DM controls [29]. Other studies have investigated flow deficits in diabetic patients [32]. These studies found that diabetic patients have a significantly higher percentage of the area occupied by flow deficits compared with controls, and this is also associated with DR severity [33]. Dimitrova et al. found that choriocapillary vessel density was decreased in diabetic patients without diabetic retinopathy compared with controls, but this difference was not statistically significant [23]. Conti et al. found that CC whole-image capillary perfusion density (CPD) decreased by 8.3% in eyes with NPDR [31]. In a study of patients with type 1 diabetes, Parravano et al. found that the average flow deficit area (FDA) was statistically significantly greater in the NPDR group compared with the NoDR group [34].

Analysis of the foveal avascular zone area showed that the mean FAZ area in SCP was 0.50 ± 0.1 mm^2^ in healthy controls (range 0.30–0.70 mm^2^) and 0.51 ± 0.1 mm^2^ in the DMnoDR group (range 0.31–0.71 mm^2^); these results are similar and show no significant difference (*p* = 0.806). On the other hand, we found that the mean FAZ area in the DMnoDR group was statistically significantly smaller than both healthy controls and the DM + DR group. This result is unexpected, considering the numerous other studies that support the theory of FAZ enlargement in diabetic patients even without retinopathy [23,35,36,37]. There may be several reasons for this reduction in the FAZ area in our mild diabetic retinopathy patients. First, we have to consider that our patients have a very early stage of diabetic retinopathy; hence, these changes in vascularity may be subtle events occurring at the beginning of the disease. This can be a sign of microvascular autoregulation in diabetes that leads to early compensatory mechanisms in retinal circulation and may also lead to increased capillary density in the foveal region to enhance retinal perfusion and nutrition. Second, increased angiogenesis, even in the absence of clinically visible retinopathy, can cause subtle vascular changes, including a slight increase in the number of capillaries in the foveal area. In diabetes mellitus, prolonged high blood sugar damages retinal microvasculature, leading to ischemia and localized hypoxia. Hypoxia stabilizes the transcription factor HIF-1α, which upregulates vascular endothelial growth factor (VEGF) expression. VEGF promotes endothelial cell proliferation, migration toward ischemic tissue, and survival and prevention of capillary dropout. Collectively, these can explain capillary remodeling and increased vessel density in the early stages of diabetic retinopathy, with the aim of reducing hypoxia and increasing perfusion. This could reduce the size of the FAZ since there is no need to maintain a larger avascular area. Finally, we must highlight that our study sample was much larger than those of the mentioned references: one hundred and seventy-five diabetic patients compared to sixty-two, thirty-nine, sixty-five, and sixty-three patients, respectively [23,35,36,37].

In line with these results, higher VD in the foveal region was observed in our patients with diabetic retinopathy in both the SCP (statistically significant) and the DCP (not statistically significant). These findings suggest that in the early stages of diabetic retinopathy, a compensatory mechanism may lead to a discrete increase in vascular density in response to hypoxia, specifically in the foveal region. On the other hand, our control group had a larger FAZ area than that reported in the literature using fluorescein angiography. For instance, Arend’s control group had an FAZ size of 0.231 mm^2^, Mansour’s 0.405 mm^2^, Bresnick’s 0.35 mm^2^, Sander’s 0.367 mm^2^, and Conrath’s 0.152 mm^2^ [5,6,7,38,39]. In a review of multiple studies, Khadamy reported that the average FAZ area in healthy controls ranged from 0.25 to 0.40 mm^2^ when measured using OCTA [40]. The relatively large FAZ measurements in our study may be explained by the older age of our control participants. For example, a study of a young healthy Irish population (aged 18–35 years) found a mean FAZ area of 0.22 mm^2^ [41].

It is important to emphasize that all three groups in our study were matched for sex, age, hypertensive status, and HbA1c values, but not for disease duration. It is well known that both HbA1C values [42] and disease duration [43] can influence vascular density. In future research, changes in capillary density should be monitored in relation to disease duration and patients’ glycemic control, and longitudinal studies should be conducted. Additionally, variability in measurement methods, technical differences across OCTA devices, and imaging artifacts may have contributed to these discrepancies.

## 5. Conclusions

Reduction in vascular density in the SCP of the macular and parafoveal regions, as well as in the macular, parafoveal, and perifoveal regions in the DCP, may serve as indicative associations suggesting the progression of diabetic retinopathy from a subclinical to a clinical stage. Decreased vascular density in the perifovea and entire macula within the SCP could be used as a screening marker in diabetic patients without clinically detectable retinopathy. However, these changes were not observed in the DCP, which may suggest that microvascular alterations initially occur in the SCP and only later affect the DCP.

Increased vascular density in the foveal region of the SCP, along with a reduction in the foveal avascular zone in DCP, may reflect an early compensatory mechanism at the onset of foveal involvement in diabetic retinopathy.

The main strength of this study lies in its large sample size and the focus on patients without clinical signs of diabetic retinopathy or those in its very early stages. Our findings may facilitate our understanding of the earliest pathophysiological vascular alterations in the macula of diabetic patients and highlight a critical point in the subtle onset of early diabetic maculopathy. This underscores the importance of early detection and monitoring as much as the identification of the earliest disease biomarkers, which could contribute to the development of preventive strategies and timely therapeutic interventions. Moreover, it facilitates the identification of patients who, although lacking clinical signs of microangiopathy, are at increased risk of developing ocular complications and therefore require more intensive endocrinological and ophthalmological follow-ups.

## Figures and Tables

**Table 1:** Vascular density in superficial capillary plexus.

	DMnoDR * (N = 175) Mean ± SD (%)	DM + DR * (N = 84) Mean ± SD (%)	Control (N = 171) Mean ± SD (%)	DMnoDR vs. Control *p* Value **	DM + DR vs. Control *p* Value **	DMnoDR vs. DM + DR *p* Value **
SCP * macula	46 ± 4.1	44.9 ± 4.2	47 ± 3.8	**0.025**	**0.000**	**0.034**
SCP fovea	18.4 ±7.8	20.6 ± 8	19.2 ± 7.7	0.308	0.177	**0.029**
SCP parafovea	47.5 ± 5.6	45.9 ± 4.9	47.8 ± 5.8	0.691	**0.013**	**0.029**
SCP perifovea	46.8 ± 4.2	45.7 ± 4.3	47.8 ± 3.8	**0.022**	**0.000**	0.051

* SCP—superficial capillary plexus; DMnoDR—diabetic participants without diabetic retinopathy; DM + DR—diabetic participants with diabetic retinopathy. ** One-way ANOVA with post hoc test of multiple comparisons.

**Table 2:** Vascular density in deep capillary plexus.

	DMnoDR * (N = 175) Mean ± SD (%)	DM + DR * (N = 84) Mean ± SD (%)	Control (N = 171) Mean ± SD (%)	DMnoDR vs. Control *p* Value **	DMnoDR vs. DM + DR *p* Value **	DM + DR vs. Control *p* Value **
DCP * macula	44.9 ± 5.4	42.5 ± 4.9	45.2 ± 4.8	0.574	**0.001**	**0.000**
DCP fovea	33.3 ± 8.7	34.0 ± 7.3	33.6 ± 8.2	0.522	0.730	0.719
DCP parafovea	51.3 ± 4.5	48.7 ± 4.3	50.9 ± 4.7	0.369	**0.000**	**0.000**
DCP perifovea	45.5 ± 6	43.2 ± 5.7	45.8 ± 5.5	0.612	**0.002**	**0.001**

* DCP—superficial capillary plexus; DMnoDR—diabetic participants without diabetic retinopathy; DM + DR—diabetic participants with diabetic retinopathy. ** One-way ANOVA with post hoc test of multiple comparisons.

**Table 3:** Flow CC and FAZ SCP.

	DMnoDR * (N = 175) Mean ± SD (mm^2^)	DM + DR * (N = 84) Mean ± SD (mm^2^)	Control (N = 171) Mean ± SD (mm^2^)	DMnoDR vs. Control *p* Value **	DMnoDR vs. DM + DR *p* Value **	DM + DR vs. Control *p* Value **
FLOW CC *	2.1 ± 0.1	2.0 ± 0.1	2.1 ± 0.1	0.143	**0.000**	**0.000**
FAZ SCP *	0.51 ± 0.1	0.33 ± 0.08	0.50 ± 0.1	0.806	**0.003**	**0.002**

* FLOW CC—flow area in choriocapillaris; FAZ SCP—foveal avascular zone area in superficial capillary plexus; DMnoDR—diabetic participants without diabetic retinopathy; DM + DR—diabetic participants with diabetic retinopathy. ** One-way ANOVA with post hoc test of multiple comparisons (FLOW CC) and Kruskal–Wallis and Mann–Whitney tests (FAZ SCP).

## Data Availability

The original contributions presented in this study are included in the article. Further inquiries can be directed to the corresponding author.

## Abbreviations

The following abbreviations are used in this manuscript:

DM	Diabetes mellitus
DR	Diabetic retinopathy
ETDRS	Early Treatment Diabetic Retinopathy Study
OCTA	Optical coherence tomography angiography
SCP	Superficial capillary plexus
DCP	Deep capillary plexus
FAZ	Foveal avascular zone
FLOW CC	Flow area in the choriocapillaris
VD	Vascular density
DM + DR	Diabetic patients with diabetic retinopathy
DMnoDR	Diabetic patients without diabetic retinopathy
NPDR	Non-proliferative diabetic retinopathy
